# Functional and Structural Consequences of Nine* CYP21A2* Mutations Ranging from Very Mild to Severe Effects

**DOI:** 10.1155/2016/4209670

**Published:** 2016-09-19

**Authors:** Débora de Paula Michelatto, Leif Karlsson, Ana Letícia Gori Lusa, Camila D'Almeida Mgnani Silva, Linus Joakim Östberg, Bengt Persson, Gil Guerra-Júnior, Sofia Helena Valente de Lemos-Marini, Michela Barbaro, Maricilda Palandi de Mello, Svetlana Lajic

**Affiliations:** ^1^Laboratório de Genética Molecular Humana, Centro de Biologia Molecular e Engenharia Genética, Universidade Estadual de Campinas, Av. Cândido Rondon 400, 13083-875 Campinas, SP, Brazil; ^2^Department of Women's and Children's Health, Karolinska Institutet, Pediatric Endocrinology Unit (Q2:08), Karolinska University Hospital, 171 76 Stockholm, Sweden; ^3^Departamento de Pediatria, Faculdade de Ciências Médicas, Universidade Estadual de Campinas, Rua Tessália Vieira de Camargo 126, 13083-887 Campinas, SP, Brazil; ^4^Science for Life Laboratory, Department of Medical Biochemistry and Biophysics, Karolinska Institutet, 171 77 Stockholm, Sweden; ^5^Science for Life Laboratory, Department of Cell and Molecular Biology, Uppsala University, 751 24 Uppsala, Sweden; ^6^Department of Molecular Medicine and Surgery, Karolinska Institutet and Center for Inherited Metabolic Diseases (CMMS L7:05) Karolinska University Hospital, 171 76 Stockholm, Sweden

## Abstract

We present the functional and structural effects of seven novel (p.Leu12Met, p.Arg16Cys, p.Ser101Asn, p.Ser202Gly, p.Pro267Leu, p.Gln389_Ala391del, and p.Thr450Met) and two previously reported but not studied (p.Ser113Phe and p.Thr450Pro)* CYP21A2* mutations. Functional analyses were complemented with* in silico* prediction of mutation pathogenicity based on the recently crystallized human CYP21A2 structure. Mutated proteins were transiently expressed in COS-1 cells and enzyme activities towards 17-hydroxyprogesterone and progesterone were determined. Residual enzyme activities between 43% and 97% were obtained for p.Arg16Cys, p.Ser101Asn, p.Ser202Gly, p.Pro267Leu, and p.Thr450Met, similar to the activities of the well-known nonclassic mutations p.Pro453Ser and p.Pro482Ser, whereas the p.Leu12Met variant showed an activity of 100%. Conversely, the novel p.Ser113Phe, p.Gln389_Ala391del, and p.Thr450Pro mutations drastically reduced the enzyme function below 4%. The *K*_*m*_ values for all novel variants were in the same order of magnitude as for the wild-type protein except for p.The450Met. The maximum velocity was decreased for all mutants except for p.Leu12Met. We conclude that p.Leu12Met is a normal variant; the mutations p.Arg16Cys, p.Ser101Asn, p.Ser202Gly, p.Pro267Leu, and p.Thr450Met could be associated with very mild nonclassic CAH, and the mutations p.Ser113Phe, p.Gln389_Ala391del, and p.Thr450Pro are associated with classic CAH. The obtained residual activities indicated a good genotype-phenotype correlation.

## 1. Introduction

Congenital adrenal hyperplasia (CAH) is caused by defects in one of the steroidogenic enzymes involved in the adrenal steroid biosynthesis from cholesterol to cortisol. The most common cause of CAH is 21-hydroxylase deficiency (21OHD) where patients with the classic form present with or without salt loss (salt wasting or simple virilizing forms, resp.) during the neonatal period and affected females are born with virilized external genitalia. In the nonclassic form, signs of androgen excess such as acne, hirsutism, and menstrual irregularities can be observed as late as during adolescence and adulthood. Children present with precocious pubarche, accelerated growth velocity, and advanced skeletal maturation [1]. The worldwide incidence of classic 21OHD is 1 : 10.000 to 1 : 15.000 live births, while nonclassic 21OHD is much more prevalent occurring in 1 : 500 live births in Caucasian populations [2–5].

The* CYP21A2* gene coding for the 21-hydroxylase enzyme is formed by 10 exons and 9 introns located on the short arm of chromosome 6 [6, 7].* CYP21A2* is arranged in tandem with a nonfunctional pseudogene (*CYP21A1P*) that shares 98% sequence identity with the active gene [8, 9]. There are common pseudogene-derived mutations identified in* CYP21A2* that are found in more than 95% of all CAH alleles. In general, the less severe mutation present in the genotype determines the phenotype, establishing a good genotype-phenotype correlation [10]. Furthermore,* in vitro* studies indicate that mutated CYP21A2 residual enzyme activities present a good correlation with* in vivo* disease severity [11–13]. Therefore,* in vitro* analysis for novel or rare mutations is proposed as a complement for disease classification, especially where large groups of patients are not available for clinical investigation, thereby improving genetic counselling and clinical management [14–17].

In this report, we describe a detailed evaluation of the functional role of seven novel (p.Leu12Met, p.Arg16Cys, p.Ser101Asn, p.Ser202Gly, p.Pro267Leu, p.Gln389_Ala391del, and p.Thr450Met) and two previously reported but not functionally studied (p.Ser113Phe and p.Thr450Pro) CYP21A2 mutations [18, 19]. The aim of the study was to investigate the pathogenicity of novel/rare mutations using* in vitro* assays and to establish a correlation between their* in vitro* effect and a possible CAH phenotype. In order to reach a reliable phenotype prediction, we also expressed four mutations (p.Ile172Asn, p.Val281Leu, p.Pro453Ser, and p.Pro482Ser) known to cause CAH of different severity, as well as a CYP21A2 normal variant (p.Ala15Thr). Furthermore, we complemented the functional analyses with* in silico* predictions of mutation pathogenicity and the effects on protein structure using the model of the recently crystallized human CYP21A2 structure [20].

## 2. Material and Methods

### 2.1. Genotyping

*CYP21A2* genotyping was performed at Universidade Estadual de Campinas, Brazil, and at Karolinska University Hospital, Sweden. The study was approved by the Ethics Committee of Universidade Estadual de Campinas and the Regional ethics committee of Karolinska Institutet. Genomic DNA was obtained from peripheral blood by phenol/chloroform extraction. The* CYP21A2 *gene was specifically amplified in two or four fragments, depending on the presence or absence of the variant C at the intron 2 c.290-13A/C>G position [21]. Sequencing of the amplified products has been performed using the BigDye Terminator v3.1 Cycle Sequencing kit (Applied Biosystems, USA) according to the manufacturer's instructions. Fragments were separated on a Genetic Analyzer from Applied Biosystems (ABI PRISM® 3100 Genetic Analyzer/Life Technologies, USA). Electropherograms were analyzed against the reference sequence NM_000500.6. Segregation analysis was performed in all subjects by sequencing parental samples, except for subject 3.

### 2.2. Subjects

Clinical and molecular data of the subjects are summarized in Table 1.

Subject number 1, a girl, presented with premature pubarche and accelerated growth velocity at the age of 4 years. She had clitoral enlargement without labioscrotal fusion since birth (Prader I). Due to advanced bone age (8.7 y at 6.4 y chronological age) she was subjected to 250 *μ*g Synacthen stimulation test that exhibited an elevation in the 17OHP basal level from 13 nM to >154 nM at 60 min. The child was diagnosed with NC CAH and* CYP21A2* genotyping revealed a complex genotype with a novel mutation p.Leu12Met together with p.Gln318^*∗*^ on the maternal allele and the NC mutation p.Val281Leu on the paternal allele.

Subject number 2 came to medical attention when her daughter was diagnosed with CAH and the family was genotyped for 21OHD. She did not complain of any symptoms of androgen excess and stimulation with Synacthen raised the 17OHP basal level just above the cut-off at 60 min (6 to 31 nM).* CYP21A2 *genotyping revealed a novel mutation, p.Ser101Asn, in compound heterozygosity with p.Val281Leu. Therefore, very mild NC CAH was suspected but was not clinically confirmed in this case.

Subject number 3, a female, presented with menstrual irregularities, hirsutism and a clinical suspicion of NC CAH at the age of 26 years.* CYP21A2* genotyping revealed the common p.Val281Leu mutation and the novel p.Ser113Phe amino acid change. Although segregation analyses or biochemical investigational data were not available, we assumed that the genotype could be responsible for her clinical presentation and NC CAH.

Subject number 4, a girl, was born appropriate for gestational age at gestational week (GW) 36 and detected via the neonatal screening program. The 17OHP screening level (106 nM) was slightly above the cut-off level (100 nM). The second tier remained elevated (66 nM, cut-off 60 nM, GW37+2). At birth she had no signs of virilization or salt loss. At 6 months of age, a 250 *μ*g Synacthen stimulation test was normal. Due to her borderline hormonal screening values* CYP21A2* genotyping was performed. The novel p.Ser202Gly mutation was identified in* trans* with p.Gln318^*∗*^. Very mild NC CAH could at this stage not be ruled out.

Heterozygosity for the p.Ser202Gly variant was also identified in subjects number 5 and number 6 at the ages of 1 month and 9 years, respectively. Both children were initially seen at a primary reference center and investigated for suspected signs of androgen excess. However, the analyses of 17OHP, genotyping, and clinical examination could not confirm the diagnosis of CAH. These subjects are thus heterozygous carriers for the novel p.Ser202Gly mutation.

Subject number 7, a girl, was virilized at birth (Prader IV) and presented with a salt-losing crisis at day 7. SW CAH was confirmed due to elevated levels of 17OHP and subsequent genotyping identified that she was compound heterozygous for the novel in-frame deletion p.Gln389_Ala319del inherited from her father and a 30-kb deletion inherited from her mother.

The detailed clinical presentation of subject number 8 has been previously described [19]. The girl was born virilized (Prader V) and presented with an adrenal crisis at day 33. She was found to be homozygous for the p.Thr450Pro mutation.

Subjects numbers 9, 10, and 11 presented with different signs of androgen excess and clinical suspicion of CAH; for summary see Table 1. However, 17OHP levels remained within the normal range even after Synacthen stimulation. Genotyping revealed that these subjects were heterozygous carriers for three novel mutations, p.Arg16Cys, p.Pro267Leu, and p.Thr450Met, respectively. Since the functional consequences for these novel amino acid substitutions were unknown, they were included in the present investigation.

### 2.3. Functional Studies

The general description for the construction of vectors used in the COS-1 expression studies of mutated CYP21A2 has been previously described [11, 22].

Expression of wild-type and mutant CYP21A2 enzymes and assays of 21OH activity were performed as previously described [23, 24]. Enzyme activities were expressed as a percentage of conversion, taking the apparent specific activity of the CYP21A2 wild type as 100%. Assays were performed after 40 min of incubation time.

Apparent kinetic constants were determined 24 h after transfection. Intact cells were incubated as previously described [23] together with 0.5, 1.0, 2.0, 3.0, or 6.0 *μ*M of unlabeled 17-hydroxyprogesterone (Sigma-Aldrich, Saint Louis, USA) as substrate. After incubation at 37°C for 20 min, steroids were extracted and analyzed as previously described [23]. Apparent kinetic constants were calculated after linear regression of the data derived from determinations of enzymatic activity at each of the five different substrate concentrations.

### 2.4. Western Blot

To ascertain similar amount of CYP21A2 expression in transfected cells, Western blotting was performed using rabbit polyclonal antibodies against human CYP21A2 as primary antibody (Sigma-Aldrich, Saint Louis, USA) and anti-rabbit IgG (GE Healthcare Life Sciences, Freiburg, Germany) as the secondary antibody; see Robins et al. [23] for details.

### 2.5. Structural Evaluation

The resolved three-dimensional structure of human CYP21, pdb id: 4y8w [18], was used as a starting point to evaluate the effect of the novel mutations on the protein structure. The ICM molecular modelling software (version 3.8-1, Molsoft LLC, La Jolla, CA) was used to perform structural calculations of the structures of each mutation, expanding on the strategy previously described [25]. First, all missing intrastructural loops were added to the 4y8w structure and the structure was optimized using energy minimization. A model of each mutation was then generated by modifying the corresponding amino acid residue, followed by multiple steps of energy minimization. The energy minimization was initially performed with strong backbone restraints, which were gradually relaxed and finally a global minimization without backbone restraints was performed. Each mutation was then evaluated based on energy and distances to the steroid and heme, as well as using the ICM built-in function for evaluating stability changes from mutations by calculating the free energy changes.

The BLAST web interface [26] was used to identify mammalian proteins similar to the human CYP21A2 enzyme. The search was performed on the Nov 2015 release of the Refseq database and resulted in CYP21 protein sequences from 81 mammals. The sequences with the highest similarity to the human CYP21A2 protein, one sequence per species, were retrieved from the database. The retrieved sequences were aligned using the Linsi approach of MAFFT [27], and for each position the sequence identity was calculated, counting a gap as a mismatch.

## 3. Results

**Figure 1:**
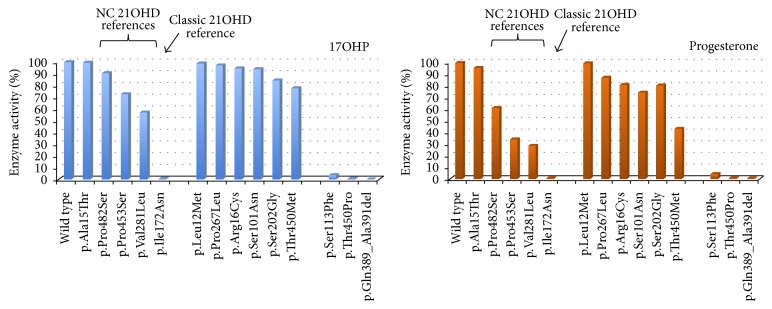
Enzymatic activities of CYP21A2 mutant proteins. Activities are expressed as a percentage of wild-type activity, which is arbitrarily defined as 100%. Conversion values are shown for the two natural substrates (17OHP and progesterone).

We have determined the residual activities of seven novel* CYP21A2* mutations (p.Leu12Met, p.Arg16Cys, p.Ser101Asn, p.Ser202Gly, p.Pro267Leu, p.Gln389_Ala391del, and p.Thr450Met) and of two previously reported but not functionally studied alterations (p.Ser113Phe and p.Thr450Pro). Neither of the variants was present in the pseudogene. We compared the data to the residual activities of five well-known reference mutations (p.Ala15Thr, p.Ile172Asn, p.Val281Leu, p.Pro453Ser, and p.Pro482Ser) using* in vitro* functional assays. The different residual enzyme activities ranged from normal to completely abolished enzyme function and the mutations could therefore be defined as being normal polymorphisms or being able to cause CAH of various degrees of severity; Figure 1 and Table 3 illustrate the results. In order to unravel the mechanism responsible for reduced enzyme function and to better differentiate between a very mild mutation and a neutral amino acid change we determined the apparent kinetic constants for all mutants that exhibited a residual enzyme activity above 80% compared to the wild-type protein (Table 2). *K*_*m*_ (substrate-binding capacity) for all variants was in the same order of magnitude as for the wild-type protein except for the p.Thr450Met mutation. The maximum velocity (*V*_max_) was decreased for all variants, except for p.Leu12Met and for the reference mutation p.Arg15Thr, and both are thus considered to be normal variants (Table 2).

Equal protein expressions of mutant and wild-type proteins have been confirmed by Western blotting (data not shown).

**Figure 2:**
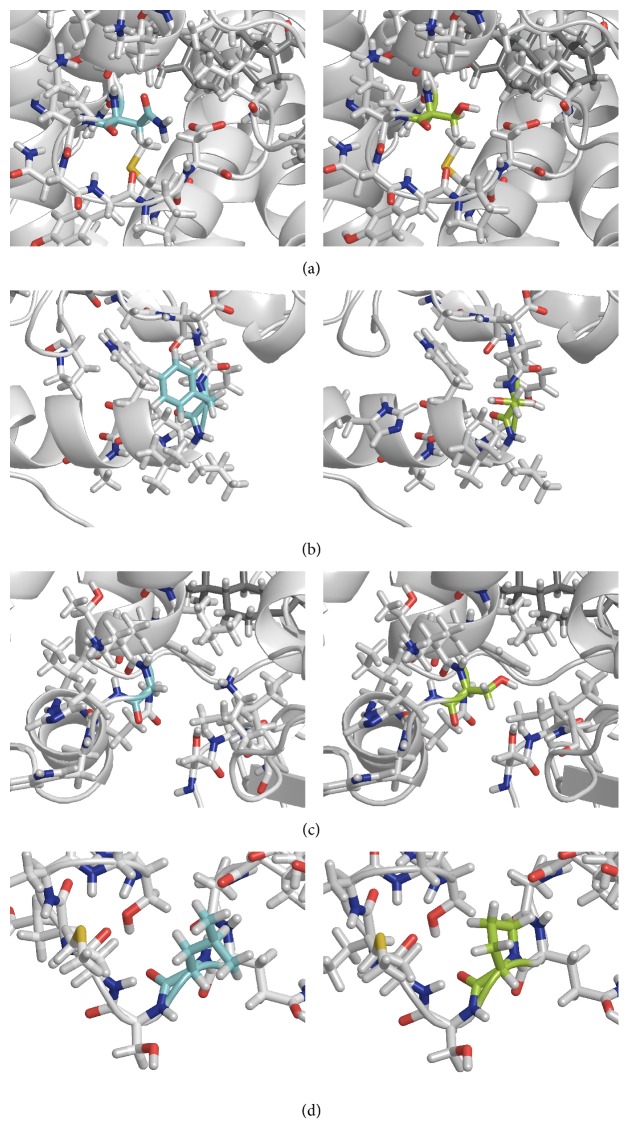
Structural changes. Visualization of the structural changes caused by four mutations; left: wild type; right: mutation; (a) p.Ser101Asn, (b) p.Ser113Phe, (c) p.Ser202Gly, and (d) p.Pro267Leu.

Table 3 summarizes the biochemical results, the putative effects on the protein structure for all mutants, and the final prediction of the phenotype. Structural calculations for p.Ser101Asn, p.Ser113Phe, Ser202Gly, and p.Pro267Leu are also illustrated in Figure 2. The p.Pro267Leu mutation has no effect on protein structure, whereas p.Ser101Asn, p.Ser202Gly, and p.Thr450Met are predicted to have a minor effect. Conversely, p.Ser113Phe produces a more severe effect on protein structure since it interferes with the alfa helices comprising the active site. The p.Thr450Pro mutation and the p.Gln389_Ala391del in-frame deletion are predicted to have deleterious effects on the CYP21A2 structure.

## 4. Discussion

Nine novel/rare mutations, identified in individuals investigated and genotyped for CAH, have been expressed* in vitro* and enzymatic activities were compared with the functional activity of the wild-type protein. Five other well-known reference mutations were included in the assay. The reference mutations present a gradient of increasing residual activities that are associated with the CAH phenotype variability. The p.Ile172Asn mutation is a classic mutation in most cases leading to SV CAH [28], with a very low residual enzyme activity towards both 17OHP and progesterone. The mutations p.Val281Leu, p.Pro453Ser, and p.Pro482Ser are three NC CAH mutations that present different degrees of residual activity, p.Pro482Ser being the mildest variant [29] and p.Val281Leu being the most frequent mutation causing NC CAH with a residual activity of approximately 50% [30]. At last, p.Ala15Thr is considered to be a neutral amino acid change [29]. Expressing these well-characterized mutations at the same time as the novel mutations allowed a better intercomparison among mutants and provided a genotype-phenotype prediction.

Two of the analyzed mutations, p.Leu12Met and p.Arg16Cys, are located in the first hydrophobic domain of the protein. This region comprises the membrane targeting, anchoring domain and is important for* in vitro* stability [31]. It is unsuitable for computational protein structure analysis and is missing from the published crystal structures [20, 32]. Leu12 is highly conserved among mammalian species (88%). Arg16 is also well conserved (69%) and the corresponding amino acid residue in bovine is histidine, indicating conservation of a positively charged, polar residue. Interestingly, another mutation within this region, p.Ala15Thr, is a normal variant [29]. The mutation p.Leu12Met was identified in* cis* with the p.Gln318^*∗*^ mutation and consequently this is a null allele, whereas the p.Arg16Cys was identified in a heterozygous carrier. Therefore it was impossible to evaluate their pathogenicity based on the phenotype of the subjects (Table 1).* In vitro* functional studies showed that the mutant p.Leu12Met did not demonstrate a significant difference in enzymatic activity or in kinetic constants when compared with the wild-type and p.Ala15Thr proteins. It is therefore considered to be a normal variant. The p.Arg16Cys mutant demonstrated a very mild reduction in enzyme activity, similar to p.Pro482Ser (Table 3) in addition to a clear reduction in apparent *V*_max_ (Table 2). Although we did not identify a compound heterozygous patient to confirm our hypothesis, this mutation may be associated with a very mild NC CAH phenotype.

The p.Ser101Asn mutation was identified in an asymptomatic mother of a Brazilian CAH patient (Table 1). She was compound heterozygous for the p.Val281Leu mutation that was transmitted to her daughter. Ser101 is conserved among mammalian species (74%) and is located between two residues that are part of the channel for product passage [32]. However, the structural calculations implied only a minor effect on protein structure caused by the mutation. In fact,* in vitro* studies confirmed a very mild effect with a reduction of activity to 94% and 74% towards 17OHP and progesterone, respectively. *V*_max_ value was also reduced as shown in Table 2. Based on our results we may expect this mutation to result in very mild NC CAH. However, we could not confirm our hypothesis because the individual did not present any obvious symptoms and a neutral amino acid change could not completely be ruled out.

The mutation p.Ser113Phe may disturb an adjacent *α*-helix based on the structural calculations for the model of human CYP21A2. It is located in the vicinity of Ser109, Leu110, and Trp117, which form part of the proximal substrate-binding pocket and the heme-binding region [32]. Therefore this amino acid substitution may interfere with the recognition and binding of the substrate and, consequently, affect enzyme activity. The* in silico* predictions were in line with the* in vitro* studies where a minimal activity of 4% towards both substrates was obtained (Table 3). Consequently, p.Ser113Phe can be associated with SV CAH if found in a homo- or hemizygous state. Although p.Ser113Phe had been previously reported by Haider et al. [18] as a NC mutation, no patient description or* in vitro* studies were presented in the first report.

The p.Ser202Gly mutation was identified in three independent subjects from two different continents. The two Brazilian carriers were genotyped because CAH was suspected but no other mutations were identified. In the Swedish subject, however, it was identified in compound heterozygosis with the null mutation p.Gln318^*∗*^. This girl has been followed during her first year of life and so far no signs of virilization have been detected, but a NC CAH phenotype cannot be ruled out at this young age. Functional studies showed a reduction in enzyme activity to the level of the NC mutation p.Pro482Ser and a reduced *V*_max_ (Tables 2 and 3). The structural calculations implied that this mutation causes a minor effect on protein structure since both serine and glycine are small amino acid residues and Ser202 is located in a loop close to the surface of the protein.

The p.Pro267Leu mutation was identified in a girl that was investigated for premature pubarche at 4 years of age.* CYP21A2* genotyping indicated that she was a heterozygous carrier for this mutation. Pro267 is weakly conserved among mammalian species (51%) and is located in a loop between two *α*-helices. The structural calculations did not show any notable structural changes. Functional studies showed a minor effect on enzyme activity similar if not milder than for p.Pro482Ser. Kinetic studies showed a reduction in *V*_max_ but to finally exclude p.Pro267Leu as a neutral variant additional and symptomatic CAH cases with this mutation need to be identified (Table 2).

The* in-frame* deletion of three amino acids (p.Gln389_Ala391del) is the result of the nucleotide deletion c.1165_1173delCAAGGCGCC in exon 9. Based on the phenotype of the patient who has SW CAH (Table 1) a drastic effect on the enzyme activity could be predicted. The patient is compound heterozygous for this mutation inherited from her father and a 30-kb deletion inherited from her mother. The three deleted residues (Glu, Gly, and Ala) are highly conserved among mammalian species (83%, 94%, and 94%, resp.). Based on the structural analysis of human CYP21A2, these amino acids are located inside an *α*-helix just before a His that has been shown to interact with the EXXR structural motif, important for the tertiary structure of the CYP21A2 enzyme [32]. The deletion by itself may be deleterious to the structure or function of the protein.* In vitro* studies confirm the loss of enzyme activity. No reduction in protein level was seen by Western blot analysis (data not shown), although specific stability studies have not been performed.

Finally, the novel mutation p.Thr450Met has been identified in a heterozygous carrier in the Brazilian population. Previously, the p.Thr450Pro mutation was identified in an Iranian girl with SW CAH (Table 1). Thr450 is highly conserved (89%) among mammalian species and is located inside a short *β* sheet, which is disturbed by the p.Thr450Pro mutation. The *β* sheet is not central to the protein, but the structural calculations imply that the mutation affects protein structure significantly. The p.Thr450Met substitution, however, does not disturb the *β* sheet to the same extent. We expressed both mutant proteins* in vitro* and obtained results that confirmed the structural predictions (Table 3). The p.Thr450Pro mutation had a drastic effect on enzyme function, similar to the classic reference mutation p.Ile172Asn, whereas the p.Thr450Met mutant gives a milder reduction in enzyme activity to the level of other NC mutations (Table 3) but with a more severe impact on apparent kinetic constants (Table 2).

## 5. Conclusions

We studied the functional effects of nine novel/rare mutations in the CYP21A2 enzyme. We conclude that p.Leu12Met is a normal variant and the mutations p.Ser113Phe, p.Gln389_Ala391del, and p.Thr450Pro are associated with classic CAH. The mutations p.Arg16Cys, p.Ser101Asn, p.Ser202Gly, p.Pro267Leu, and p.Thr450Met could be associated with milder forms of nonclassic CAH, although the identification of additional symptomatic cases will further define the clinical spectrum for these variants. The study showed a good correlation between genotype and phenotype for mutations identified in compound heterozygous individuals. Furthermore, the putative effects on protein structure based on* in silico* predictions using the recently crystallized human CYP21A2 structure confirmed the pathogenicity of the mutations and the results were in agreement with the functional studies.

## Figures and Tables

**Table 1:** The genotypes and symptoms at diagnosis of the individuals carrying the novel mutations (marked in bold).

Subject number	Sex	Age at diagnosis	Symptoms	17OHP (nmol/liter)^*∗*^ basal/stimulated	Genotype	Phenotype	Country of origin	Publication
1	F	4 years	Prader I, premature pubarche, accelerated growth	13/>154	**p.Leu12Met**+p.Gln318^*∗*^/p.Val281Leu	NC	Brazil	Present study
2	F	Adult	Asymptomatic	6/31	**p.Ser101Asn**/p.Val281Leu	—	Brazil	Present study
3	F	Adult	Menstrual irregularities and hirsutism	NA	**p.Ser113Phe**/p.Val281Leu	NC	Brazil	Present study^*∗∗∗*^
4	F	At birth	Positive neonatal screening, 17OHP 106 nM^*∗∗*^	5/21 Cortisol 360/1118	**p.Ser202Gly**/p.Gln318^*∗*^	—	Sweden	Present study
5	F	1 month	Prader I, screening value 17OHP 23 nM	NA	**p.Ser202Gly**/WT	Healthy carrier	Brazil	Present study
6	M	9 years	Premature pubarche, advanced BA (12.1 y at 9.5 y)	4/11	**p.Ser202Gly**/WT	Healthy carrier	Brazil	Present study
7	F	At birth	Prader IV, Na (129 mM)	73/NA	**p.(Gln389_Ala391del)**/30-kb deletion	SW	Brazil	Present study
8	F	1 month	Prader V. Adrenal crisis day 33: Na (117 mM), K (8.3 mM), hypoglycemia (2.3 mM)	247/NA	**p.Thr450Pro/p.Thr450Pro**	SW	Iran	[19]
9	M	5 years	Suspicion of penile growth/pseudoprecocious puberty	1.5/NA	**p.Arg16Cys**/WT	Healthy carrier	Brazil	Present study
10	F	4 years	Premature pubarche, BA 8.10 yrs at 6.5 yrs of age	2/7	**p.Pro267Leu**/WT	Healthy carrier	Brazil	Present study
11	F	Adult	Acne	1.3/9	**p.Thr450Met**/WT	Healthy carrier	Brazil	Present study

^*∗*^Reference value/cut-off level 17-OHP: 6 and 30 nM, basal and 60 min, 250 *µ*g Synacthen stimulation test.

^*∗∗*^Neonatal reference value/cut-off level 100 nM.

^*∗∗∗*^Haider et al. 2013 [18], mutation is reported but no patient phenotype description.

NA: not available; SW: salt wasting; NC: nonclassic; BA: bone age; WT: wild type.

**Table 2:** Apparent kinetic constants using 17-OHP as substrate.

	*V*_max_ (pmol/mg protein per min)	*K*_*m*_ (*µ*M)	*V*_max_/*K*_*m*_
Wild type	519 (93)	12.5 (1.3)	42 (9)
p.Leu12Met	618 (207)	17.7 (5.2)	35 (4)
p.Ala15Thr	671 (145)	16.9 (6.5)	42 (8)
p.Arg16Cys	181 (31)	5.0 (1.1)	37 (3)
p.Ser101Asn	326 (67)	9.7 (2.0)	34 (4)
p.Ser202Gly	244 (47)	8.0 (1.5)	31 (5)
p.Pro267Leu	320 (93)	8.4 (1.9)	38 (7)
p.Thr450Met	43 (5)	1.2 (0.1)	35 (5)

Values are present as the mean (1SD) of at least four experiments.

**Table 3:** Enzyme activities, *in silico* prediction of the effect on protein structure, and final prediction of the phenotype for the novel mutations and for the common mutations used as a reference (in bold).

Mutation (cDNA)	Protein change	Location in the protein	*In silico* prediction	Enzyme activity		Phenotype prediction (hemizygous/homozygous state)
				17OHP	Prog	
**c.43G>A**	**p.Ala15Thr**	**First hydrophobic domain**	**ND**	**100 (0)**	**96 (6)**	**Normal variant (NV)**
c.34C>A	p.Leu12Met	First hydrophobic domain	ND	99 (1)	100 (1)	NV
c.800C>T	p.Pro267Leu	Loop between *α*-helix H and *α*-helix I	No effect	97 (1)	87 (7)	NV/very mild NC
c.46C>T	p.Arg16Cys	First hydrophobic domain	ND	95 (3)	81 (3)	NV/very mild NC
c.301_302TC>AA	p.Ser101Asn	Loop between *α*-helix B′ and *α*-helix C	Minor effect	94 (3)	74 (2)	NV/very mild NC
**c.1444C>T**	**p.Pro482Ser**	**β**-sheet** ** **β**9	**ND**	**91 (6)**	**61 (6)**	**Very mild NC**
c.604A>G	p.Ser202Gly	Loop between *α*-helix F and *α*-helix F′	Minor effect	85 (2)	81 (3)	Very mild NC
c.1349C>T	p.Thr450Met	*β*-sheet *β*8	Minor effect	78 (6)	43 (5)	Mild NC
**c.1357C>T**	**p.Pro453Ser**	**β**-sheet** ** **β**8	**ND**	**73 (8)**	**34 (10)**	**NC**
**c.841G>T**	**p.Val281Leu**	**α**-helix** I**	**ND**	**57 (8)**	**29 (5)**	**NC**
c.338C>T	p.Ser113Phe	Loop between *α*-helix B′ and *α*-helix C	Severe effect; interferes with active site helices	4 (1)	4 (2)	CL
c.1348A>C	p.Thr450Pro	*β*-sheet *β*8	Very severe effect	<1	<1	CL
**c.515T>A**	**p.Ile172Asn**	**α**-helix** E**	**ND**	**<1**	**<1**	**CL**
c.1165_1173delCAAGGCGCC	p.Gln389_Ala391del	*α*-helix L	Deleterious	0	<1	CL

Enzyme activity is expressed in % of wild-type activity, with values presented as mean (1SD) of at least four independent experiments. ND: not determined. Mutations are ordered according to the residual enzyme activity with the reference mutations in bold.
